# 2024 Advances in Learning Health System Sciences (AiLHSS) Conference: Abstracts

**DOI:** 10.1002/lrh2.70000

**Published:** 2025-02-17

## Genevieve B. Melton^1,2,3^, Andy Oien^1^, Amanda Trofholz^1^, Lindsay Bork Nichols^1^, Rui Zhang^1,2^, Mary Butler^1,4^, Sallee Brandt^1,4^, Julia Tang^1^, Timothy Beebe^1^

### ^1^Center for Learning Health System Sciences, University of Minnesota, Twin Cities, Minneapolis, MN, USA; ^2^Department of Surgery, University of Minnesota, Twin Cities, Minneapolis, MN USA; ^3^Institute for Health Informatics, University of Minnesota, Twin Cities, Minneapolis, MN USA; ^4^School of Public Health, University of Minnesota, Twin Cities, Minneapolis, MN USA

**Corresponding Author:** Timothy Beebe, beebe026@umn.edu.

## CONFERENCE SUMMARY

The *Advances in Learning Health System Sciences* (*AiLHSS) Conference* is an annual event that addresses pressing and emerging Learning Health System (LHS) topics while fostering collaboration among researchers advancing the field presented by the University of Minnesota (UMN) Center for Learning Health System Sciences (CLHSS), a collaboration between the Medical School and School of Public Health at the UMN. The 2024 conference was held on September 9–10 at Coffman Memorial Union at UMN in Minneapolis, MN. This year's conference featured 219 attendees including clinicians, researchers, policymakers, and trainees, with local, national, and international representation from professional organizations, research institutes, healthcare organizations, and universities serving the needs of diverse populations such as children, the elderly, rural communities, and veterans. The agenda was composed of keynote presentations, scientific forums, workshops, and poster sessions all designed to foster collaboration and innovation in the field of LHS. Throughout the conference, attendees had the opportunity to engage in various networking sessions. This year's agenda highlighted the importance of collaboration and continuous learning in improving healthcare delivery, with a focus on practical tools, real‐world examples, and the integration of patient and community perspectives.

On the first day, September 9, the conference began with coffee and networking, followed by welcome and opening remarks from Genevieve Melton‐Meaux, MD, PhD (Director, CLHSS), and Jakub Tolar, MD, PhD (Dean, UMN Medical School). The morning sessions included a keynote by Sarah Greene, MPH, from the National Academy of Medicine discussing the complexities and opportunities in building and sustaining learning health systems. This was followed by a spotlight session and a panel on active data sharing collaboratives. The afternoon featured scientific forums on topics such as organ donation data, opioid substance use treatment, and cardiac imaging in stroke evaluation, as well as a workshop on partnering with patients to improve study design and outcomes. The day ended with a poster session which included networking opportunities.

The second day, September 10, started with coffee and networking, followed by opening remarks from Timothy Beebe, PhD (Deputy Director, CLHSS), and a keynote by Sara Singer, PhD, MBA, from Stanford University on the importance of learning in health systems. The morning sessions included evidence synthesis case studies and a spotlight session on implementation science reporting and adherence challenges. The conference concluded with a keynote by Angela D. Thomas, DrPH, MPH, MBA, from MedStar Health discussing the realities of being a learning health system.

This article features a curated collection of abstracts from spotlight sessions, oral presentations, and the highest‐scored posters, all of which were recognized for their innovative contributions to LHS research and practice. These presentations emphasized co‐creation of evidence‐based interventions, real‐world data utilization, and systems thinking to improve patient care and outcomes. By focusing on core LHS principles such as iterative learning, stakeholder engagement, and data‐driven decision‐making, they offer practical models for addressing diverse medical and public health challenges.

## FLEXIBLE SYNTHESIS OF CATEGORICAL SEQUENCES USING PAIRED‐MC

### Zuofu Huang^1^; Julian Wolfson, PhD^1^ ; Jayne A. Fulkerson, PhD^2^ , Ryan Demmer, PhD^3^ ; Helen N. Chen, PhD^4^

#### ^1^School of Public Health, University of Minnesota, Twin Cities, Minneapolis, Minnesota, USA; ^2^School of Nursing, University of Minnesota, Twin Cities, Minneapolis, Minnesota, USA; ^3^Mayo Clinic, Rochester, Minnesota, USA; ^4^School of Public Health, Indiana University, Indianapolis, IN, USA

**Corresponding Author:** Zuofu Huang, huan2357@umn.edu.

**Introduction:** Over the last few decades, an increasing amount of data are being collected using mobile and wearable devices which enable the recording of physical and situational measures on a near continuous basis. For example, mobile apps can use contextual data on location, speed, etc. to infer human activity episodes (home, work, car, bus, etc.); the resulting data can be applied in areas from urban planning to public health. The ability to synthesize such data in a realistic and parametrizable way is valuable for a number of reasons, including privacy, missing data imputation, and evaluating the performance of statistical and computational methods. When the underlying data generating process is complex, data synthesis requires approaches that balance realism and simplicity.

**Methods:** We address the problem of synthesizing sequential categorical data of the type that is increasingly available from mobile applications and sensors that record participant status continuously over the course of multiple days and weeks. We propose the paired Markov Chain (paired‐MC) method, a flexible framework that produces sequences that closely mimic real data while providing a straightforward mechanism for modifying characteristics of the synthesized sequences.

**Results:** We demonstrate the paired‐MC method on two datasets, one reflecting daily human activity patterns collected via a smartphone application and one encoding the intensities of physical activity measured by wearable accelerometers. In both settings, sequences synthesized by paired‐MC better capture key characteristics of the real data than alternative approaches.

**Conclusion:** Paired‐MC is a novel method used to efficiently synthesize long categorical sequences that are parametrizable and realistic. In contrast to Markov Chain‐based methods, it relaxes the key Markovian property assumption and offers the possibility to account for higher‐order dependencies on lengthy sequences without sacrificing much computational performance. Our examples on human activity sequences and NHANES accelerometer data demonstrate that paired‐MC and pre‐clustering work on different types of sequential categorical data.

## MAP TO HEALTH: A PROOF‐OF‐CONCEPT PILOT TRIAL OF A DIGITAL PHYSICAL ACTIVITY INTERVENTION FOR ADULTS IN MIDLIFE

### Stephanie A. Hooker, PhD, MPH^1^ ; A. Lauren Crain, PhD^1^ ; Jule M. Muegge, MPH^1^ ; Rebecca C. Rossom, MD, MS^1^ ; Nicolaas P. Pronk, PhD^1^ ; Dhavan Prasad Pasumarthi, BTech^1^ ; Gopikrishna Kunisetty, MCA^1^ ; Kevin S. Masters, PhD^2^

#### ^1^Research and Evaluation Division, HealthPartners Institute, Minneapolis, MN USA; ^2^Department of Psychology, University of Colorado Denver, Denver, CO USA

**Corresponding Author:** Stephanie Hooker, stephanie.a.hooker@healthpartners.com.

**Introduction:** Physical activity (PA) is critically important for health and well‐being, yet more than half of adults are insufficiently active. Digital interventions to improve health behaviors are often offered as part of health plans' health promotion activities, but few of these programs are explicitly designed with health behavior theory in mind. Using the NIH Science of Behavioral Intervention Framework, this study's purpose was to determine whether the Meaningful Activity Program (MAP to Health), a mHealth PA intervention grounded in self‐determination theory that promotes meaning salience and supports basic psychological needs of autonomy, competence, and relatedness, was associated with changes in the hypothesized mechanisms of behavior change.

**Methods:** This study used a single‐arm, double baseline proof‐of‐concept pilot trial design. Patients and employees of the health system, who were adults in midlife, insufficiently active, and interested in increasing PA, were recruited for this intervention through mailed letters and online advertisements. After a 4‐week baseline period, participants engaged in MAP to Health for 8 weeks. Participants completed measures of the hypothesized mechanisms of behavior change, including meaning salience, needs satisfaction, and autonomous motivation at pretest (−4 weeks), baseline (0 weeks), midpoint (4 weeks), and posttest (8 weeks). Mixed models were used to compare changes in slope during the intervention to pre‐intervention.

**Results:** Participants included 35 adults (mean age = 50 years; 77% female; 66% white, 20% Asian, 9% Black, 6% other race). Most (91%) engaged in MAP to Health for ≥5/8 weeks. None of the hypothesized mechanisms changed significantly during the pre‐intervention phase (*p*s >0.05, *d*s <0.15). However, autonomy (*p* < 0.001, *d* = 0.76), competence (*p* < 0.001, *d* = 0.65), relatedness (*p = 0*.004, *d* = 0.46), autonomous motivation (*p* < 0.001, *d* = 0.34), and meaning salience (*p* < 0.001, *d* = 0.40) increased significantly during the intervention. Comparison of slopes to pre‐intervention revealed that increases during the intervention were significantly greater for autonomy (*p* = 0.002), competence (*p* < 0.001), and meaning salience (*p* = 0.001); however, the slopes were not significantly different for relatedness and autonomous motivation (*p*s ≥0.10).

**Conclusion:** MAP to Health was associated with improvements in the target mechanisms of behavior change. This is the first intervention to use meaning as a behavior change strategy in a PA intervention, and future research will test the efficacy of the intervention in increasing PA compared with a control condition. Health systems and health plans could consider offering digital interventions explicitly designed and tested using behavioral science theory and evidence to increase the likelihood that patients and members adopt behavior changes.

## DATA MODERNIZATION INITIATIVE AND PUBLIC HEALTH INFORMATICS PROJECTS AT THE MINNESOTA DEPARTMENT OF HEALTH

### Sripriya Rajamani, MBBS, PhD, MPH, FAMIA^1,2^; Kristin Sweet, PhD, MPH^3^; Aasa Dahlberg Schmit, BSc^4^; Sarah Solarz, MPH^3^

#### ^1^ Informatics Program, School of Nursing, University of Minnesota, Minneapolis, Minnesota, USA; ^2^Institute for Health Informatics, University of Minnesota, Minneapolis, Minnesota, USA; ^3^Minnesota Department of Health, Saint Paul, Minnesota, USA; ^4^HLN Consulting LLC, Mission Viejo, California, USA

**Corresponding Author:** Sarah Solarz, sarah.solarz@state.mn.us

**Introduction:** The data modernization initiative (DMI) is a multi‐billion dollar effort to develop an integrated, real‐time public health data and surveillance architecture. This national effort led by the Centers for Disease Control and Prevention (CDC) aims to modernize the data systems in public health across federal and state levels. The DMI priority areas are: (1) building the right foundation (e.g., real‐time data, automation); (2) accelerating data into action (e.g., interoperability, forecasting, analytics); (3) developing a state‐of‐the‐art workforce (e.g., recruitment, training); (4) supporting and extending partnerships (e.g., policies for data exchange and use, research/academic collaborations); (5) managing change and governance (e.g., change management). The Minnesota Department of Health (MDH) has adopted a structured DMI approach with priority projects in public health informatics. The intention is to synthesize learnings from projects and lay foundation for a learning health systems approach.

**Methods:** The DMI core team at MDH (*n* = 7) comprises the two co‐directors along with program, policy, and informatics experts. This team includes an informatics faculty from the University of Minnesota (UMN) who is an embedded researcher and supports evaluation and dissemination. The team has been meeting weekly for the past 14 months and coordinates with other relevant efforts at MDH such as the Data Vision and Roadmap project and the Public Health Infrastructure Grant (PHIG). The team has been successful in completing the first iteration of an agency‐wide workforce assessment and creating the first draft of the DMI Plan.

**Results:** The DMI approach at MDH and plan is organized under 5 pillars: 1. Planning and Coordination of DMI Activities; 2. Enhanced Workforce Capacity and Competencies; 3. Data and Public Health Information Systems Modernization; 4. Advanced Data Analysis, Reporting and Visualization Capabilities; and 5. Enhanced Data Interoperability and Data Exchanges. Each pillar has milestones and implementation time frames. The DMI team had identified 13 public health informatics projects for an LHS approach, including projects on systems interoperability between the MDH and healthcare/local public health (*n* = 6), systems modernization for programs within MDH (*n* = 5), informatics workforce development (*n* = 1), and overarching program governance (*n* = 1). Each project has been evaluated and/or has current/future assessment plans to gather lessons learned and create a feedback loop for iterative improvement. The UMN partnership's productivity is showcased with shared authorship in 10 peer‐reviewed proceedings/publications, 22 presentations, and 16 posters across local/national conferences.

**Conclusion:** The data modernization efforts at MDH present a great opportunity to institutionalize a learning health systems approach. While the LHS model to demonstrate learning and evaluation is a promising approach with case studies across healthcare delivery settings, few public health agencies today provide tangible demonstrations and partnerships across academic‐public health are fewer. Becoming a learning health system is a journey, and MDH needs to address many factors (leadership, coordination, education, partnership, dissemination, sustainability) identified by the core DMI team. In addition, there is a need to identify a LHS champion(s) and a determination to address these factors to continue this path.

**Acknowledgements:** The authors would like to thank the DMI core team and the colleagues at the Minnesota Department of Health who contributed to the various data modernization initiative projects and the MDH leadership for their support.

## A RAPID REVIEW INFORMS LEARNING HEALTH SYSTEM'S INSTITUTIONAL GUIDELINES FOR CURATIVE TREATMENT OF RESECTABLE NONMETASTATIC SOFT TISSUE SARCOMA IN TREATMENT NAÏVE PATIENTS

### Romil R. Parikh, MBBS, PhD, MPH^1^ ; Sallee Brandt, MPH^1^ ; Amy Claussen, MLIS^1^ ; Kaia Verich, RN, BSN, OCN^2^ ; Edward Cheng, MD^2^ ; Mary Butler, PhD, MBA^1^

#### ^1^ Minnesota Evidence‐Based Practice Center, University of Minnesota School of Public Health, Minneapolis, MN, USA. ^2^M Health Fairview System and Department of Orthopedic Surgery, University of Minnesota Medical School

**Corresponding Author:** Romil Parikh, parik075@umn.edu

**Introduction:** To assist the Bone and Soft Tissue Disease team in updating the current practice guidelines at the University of Minnesota, we conducted a rapid review of literature regarding the initial treatment of resectable nonmetastatic soft tissue sarcoma (STS). We targeted three key questions (KQ) framed by the clinician team, to (KQ1) compare the benefits and harms of preoperative versus postoperative radiation therapy (RT); (KQ2) compare the benefits and harms of chemoradiation versus other treatment modalities; and (KQ3) compare wound complication rates between different RT modalities.

**Methods:** We developed a search strategy that focused on perioperative strategies in a broad population undergoing surgery for curative treatment of resectable nonmetastatic STS. We screened citations from the Medline database published between 2000 and current using PICO Portal, an artificial intelligence (AI)‐enabled software. Based on the PICOTS framework, our selection criteria were: Population—adults (age, ≥18 years) with nonmetastatic, primary, resectable STS, excluding visceral, mediastinal, head, and retroperitoneal STS; Intervention and Comparator—stated in the KQ; Outcomes—all patient‐related direct outcomes for KQ1 and KQ2, and wound complication rate for KQ3; Timing—no outcome timing exclusion; **S**tudy design—most recent and most inclusive systematic reviews (SR) and primary studies on relevant points not covered in SRs. We assessed study quality using the AMSTAR tool for systematic reviews, the RoB2 tool for randomized controlled trials, and the ROBINS‐I tool for observational studies. We summarized the findings qualitatively and discussed the applicability (generalizability) of these findings. We presented our findings at the STS retreat convened to discuss updating the institutional treatment protocol/guideline.

**Results:** We conducted the review within 3 weeks. The AI‐enabled software reduced screening time by >65%. Of 1125 unique citations, we included 11 SR (moderate to critically low quality) and 13 observational studies (very low quality). We found four SR for KQ1 which reported mixed findings for efficacy, but all SR reported a higher wound complication rate in preoperative RT compared with postoperative RT and no evidence for patient‐reported outcomes. We found three poor‐quality primary studies for patient‐reported outcomes, two of which reported some evidence favoring postoperative RT over preoperative RT; however, these studies were old and used older RT modalities. For KQ2, we found three SR and 10 primary studies, all with a very high risk of bias. Histologically tailored neoadjuvant chemotherapy is likely not more effective than standard neoadjuvant chemotherapy. Evidence was insufficient for comparing combined chemoradiation with other regimens. For KQ3, we found four SR (critically low quality) with mixed findings comparing wound complication rates between RT modalities. Some studies favored newer techniques such as SBRT, IMRT, and image guided RT over conventional RT modalities, and some reported no significant difference. Owing to serious study limitations, evidence is insufficient and inconclusive.

**Conclusion:** Findings from our independent rapid review facilitated robust discussions and assisted the clinician team to: (1) update institutional practice guidelines for STS treatment and (2) identify evidence gaps to inform prioritization of research questions for their team's research development goals.

**Acknowledgements:** The authors would like to thank the Bone and Soft Tissue Sarcoma Team at the M Health Fairview System and the University of Minnesota Medical School.

## USING A LEARNING HEALTH SYSTEMS APPROACH TO BUILD A CARE COORDINATION PROGRAM FOR FAMILIES AND CHILDREN LIVING WITH CEREBRAL PALSY

### Anna Beckstrom, MS^1^ ,Kari Kubiatowicz, MBA, BSN, RN^1^ , Andrea Bushaw, PhD, APRN, CPNP^1^ , Matthew Witham, PhD, LMFT^1^ , Meghan Munger, PhD, MPH^1^

#### 1. Gillette Children's Specialty Healthcare, St. Paul, MN, US

**Corresponding Author:** Meghan Munger, meghanmunger@gilettechildrens.com

**Introduction:** Cerebral palsy (CP) is a lifelong complex condition requiring multiple difficult‐to‐navigate health system interactions. Barriers to care lead to inequities, diminished wellbeing, or poor experience. To reduce these unwanted outcomes, we developed a medical‐social model of care coordination (MSCC) and implemented it using a Learning Health Systems (LHS) framework. We aimed to quantify care coordination's impact on patients, families, and staff and understand required resources to scale and sustain.

**Methods:** We thought through: how will staff know what an individual family needs, how will leaders know what resources to provide the program, and how can we aggregate information for stakeholders (e.g., payers to influence financial sustainability).

**Results:** *Research‐to‐evidence*: Literature and internal experience influenced our outcomes selection: (1) MSCCs are effective for improving outcomes in medically‐complex children (+/− CP), (2) social determinants of health influence health outcomes, (3) evidence‐based care pathways are difficult to implement, and (4) care coordination is underfunded by payers. These factors informed our outcomes selection: patient‐reported outcomes and social risk screening (PROs), care pathway adherence, healthcare utilization, access, and experience. Our organization's explicit dedication to improving CP care prioritized new resources, including patient‐facing roles and administrative partners in operations, implementation science, and data analytics.

*Evidence‐to‐practice*: We identified eligible families using a data‐driven approach and tested demographics across enrollment waves to evaluate equity in our implementation approach. We used behavior change models to support implementation of new workflows across new and existing clinical staff. PROs guide nurse‐ and social work‐led conversations with families and are an aggregated outcome. We prioritized individual behavior change to understand barriers and facilitators to implementation of new workflows across new and existing clinical staff.

*Practice‐to‐data*: We focused on reliability and timeliness of data for operational dashboards, quality checks, and preparation for subsequent analyses. We iterated with nursing staff to ensure clinical documentation generated data conducive to analysis. We assessed staff experience and wellness to understand impacts.

**Conclusion:** Applying LHS frameworks to the development of an MSCC is valuable but presents unique challenges. “What” to implement into practice was easier, guided by literature and internal experience (*research‐to‐evidence*). However, simply having evidence that MSCC benefits patients and families is insufficient to implement, scale, and sustain within a system. *Evidence‐to‐practice* and *practice‐to‐data* components remain the bulk of current work. Behavior change models continue to guide our strategies for clinician behavior change as we prepare for broader system change. We continue to iterate on clinical documentation, data output, and analysis. These efforts underscore electronic medical record limitations in monitoring groups of patients across time. Efforts are further challenged by MSCC's inadequate payer reimbursement. Alignment with strategic initiatives opens access to organizational resources. We remain committed to bringing data on program outcomes—and the necessary resource (e.g., non‐patient‐facing time) to deliver this level of care—to payers to inform new payment strategies for MSCC. Ultimately, our commitment to applying LHS principles has given us confidence we will generate knowledge for the literature, payers, and ourselves—leading to continuous improvements in quality for our patients and families.

## STANDARDIZED INPATIENT TELESTROKE TO IMPROVE ACCESS TO STROKE SPECIALISTS

### Beatriz Rios^1^, MD*; Pramit Jagtap^1^; Samuel Boes^1^; Solmaz Ramezani Hashtjin^1^, MD; Maulik Lathiya^1^, MBBS; Saketh Annam^1^, MD; Michael Usher^1^, MD, PhD; Chloe Botsford^1^, MPH; Deborah Pestka^1^, PharmD, PhD; Joseph Koopmeiners^1^, PhD; Tanvi Mehta^1^, Timothy Beebe^1^, PhD, Genevieve Melton^1^, MD, PhD, Christopher Streib^1^, MD, MS

#### ^1^ University of Minnesota, Twin Cities, Minneapolis, Minnesota, USA

**Corresponding author:** Beatriz Rios, perei153@umn.edu

**Introduction:** Stroke is a leading cause of death and disability in the U.S., with a 10%–20% risk of recurrence in the first 90 days. Rapid access to stroke care leads to a more thorough evaluation and reduces the recurrence risk with prompt treatment. Limited availability of stroke specialist care results in suboptimal secondary stroke prevention, requires long‐distance transfers, and higher rates of stroke‐related complications. Stroke care delivered remotely by a vascular neurologist (“telestroke”) can improve specialist access and evaluation, reduces transfers, and lowers recurrence risk. We implemented inpatient telestroke management for all acute ischemic stroke (AIS) patients at five stroke‐ready hospitals in the M Health Fairview (MHFV) hospitals.

**Methods:** We compared AIS care at five stroke‐ready MHFV hospitals before and after 24/7 inpatient telestroke implementation, built in two phases: Pre‐telestroke, where in stroke care was provided by an in‐hospital staff hospitalist, and post‐telestroke, where in stroke care was provided remotely by the stroke neurology team from the University of Minnesota. Best practices for AIS care were based on the 2019 American heart Association (AHA) guidelines, and baseline data were compiled from the MN Get With the Guidelines (GWTG) stroke database and manual data abstraction. Evaluated stroke specialists' access and evaluation, transfer rates, 30/90‐day rates of readmission, 30/90‐day recurrent stroke. Utilized a stepped‐wedge cluster design to assess pre‐ and post‐telestroke intervention outcomes, adjusting for time‐ and site‐specific trends.

**Results:** A total of 1295 patients with acute ischemic stroke (AIS) were analyzed, comprising 537 before the implementation of inpatient telestroke and 758 after. The median age was 75.24 years (IQR: 64.45–86.11), with 47.7% of patients being female and 92.2% identifying as white. The median National Institutes of Health Stroke Scale (NIHSS) score was 2 (IQR: 0–5). The transfer rates before and after the implementation of inpatient telestroke were 58.5% and 37.5%, respectively, with an adjusted p‐value of less than 0.01. Among specific transfer reasons, the need for stroke specialist evaluation decreased significantly (7.4%–0.9%), and the necessity for a higher‐level stroke center also showed a notable reduction (27.6% to 18.3%). Furthermore, the rate of stroke specialist consultations increased from 80.2% to 96.4% following inpatient telestroke implementation (adjusted *p*‐value <0.01), and the thoroughness of stroke consultations improved dramatically, with full telestroke consultations rising from 1.4% to 94.1%.

**Conclusion:** Inpatient telestroke consultations that follow standard care significantly enhanced access to stroke specialists and lowered the transfer rate for acute ischemic stroke at spoke hospitals. This model of stroke care delivery was effective and could contribute to reducing persistent healthcare disparities in underserved communities.

## UNDERSTANDING PATIENT PORTAL UTILIZATION, IDENTIFYING GAPS, AND TESTING NEW STRATEGIES TO ENHANCE ADOPTION AND SUSTAINED USE FROM A HEALTH EQUITY LENS

### Mariam Hassan^1^; Zachary Henderson BSB^9^; Elizabeth Lindemann MHA^3,9^; Richard Lodahl PMP, CSM^9^; Anne Marie Hotop MS, MA, MPH^4^; Kristin Boman, MPH^6^; Jeremy Lopez BA^9^; Pita Adam MD^6^; Genevieve Melton MD PhD^2,3,5,9^; Michele Allen MD^6,7,8^; Rubina Rizvi MD PhD^2,3^

#### ^1^College of Liberal Arts, University of Minnesota, Twin Cities, Minneapolis, MN, USA; ^2^Department of Surgery, University of Minnesota, Twin Cities, Minneapolis, MN, USA; ^3^Center for Learning Health System Sciences, University of Minnesota, Twin Cities, Minneapolis, MN, USA; ^4^School of Public Health, University of Minnesota, Twin Cities, Minneapolis, MN, USA; ^5^Institute for Health Informatics, University of Minnesota, Twin Cities, Minneapolis, MN, USA; ^6^Department of Family Medicine and Community Health Primary Care Research and Learning Network, University of Minnesota, Twin Cities, Minneapolis, MN, USA; ^7^Department of Family Medicine and Community Health Program in Health Disparities Research, University of Minnesota, Twin Cities, Minneapolis, MN, USA; ^8^Center for Chronic Disease Reduction and Equity Promotion Across Minnesota, University of Minnesota, Twin Cities, Minneapolis, MN, USA; ^9^Fairview Health Services, Minneapolis, MN, USA

**Corresponding Author:** Mariam Hassan, hass678@umn.edu

**Introduction:** Patient portal (PtPl) usage has shown many such as enhanced patient engagement, improved patient satisfaction, and better quality of care. However, there are many barriers tied to PtPl usage, including socioeconomic factors. The purpose of this study is to further understand PtPl usage data from diverse groups of patient populations with chronic diseases, identify gaps, and generate intervention(s) that could bolster PtPl adoption and sustained use and ultimately improve health outcomes. This project has three specific aims across two phases (See Figure 1 below). The Exploratory Phase [Phase I] has two aims: Aim 1, to characterize usage patterns of PtPl leveraging existing EHR PtPl usage data at the institutional level, and Aim 2, to explore the perspective of patients towards PtPl utilization through data collected directly from the end‐users, that is, patients. The Interventional Phase [Phase 2] comprised Aim 3 entails the development and testing of new strategies generated from knowledge from Phase 1.

**Methods:** An embedded mixed‐methods design will be used to collect data for this study.

Aim 1: We will pull existing data from patients seen at M Health Fairview (MHFV), primarily from the Epic Clarity database, leveraging the Learning Health Systems team at MHFV. We will pull PtPl user action data across patients (50 years or older) diagnosed with one or more chronic conditions.

Aim 2: We will collect and analyze patients' perspectives towards PtPls (through surveys and interviews) from two sets of patients: (a) patients seen at MHFV Primary Care Service Line clinics meeting the above inclusion criteria, (b) Minnesota State Fair attendees who are 18 years old or older and able to complete a survey in English. Patients (or their proxy) may participate regardless of their level of usage of PtPls.

Data analysis: For quantitative data, descriptive statistics will be used. We will use linear regression models to explore the association between sociodemographic factors and PtPl use. For qualitative evaluation, we will be coding interview transcripts and survey questions leveraging a thematic analysis approach.

Aim 3: We will design and test an intervention to boost PtPl adoption and sustained use. We will measure the impacts of new interventions on patients' understanding of utility, acceptance, satisfaction, and adoption rates around PtPls. We will employ a patient participatory research approach throughout the project, and patients will be compensated for their efforts.

**Results:** Given preliminary data from participating sites, we anticipate a diverse set of participants to be enrolled in this study. Our target population is primarily individuals who may be experiencing barriers to accessing and using PtPls. Some factors we will consider within our patient population are internet access, technology access (including smartphones, computers, etc.), health literacy, socioeconomic status, and ethnicity.

**Conclusion:** We expect our project will assist us in understanding what barriers exist for patients when attempting to utilize PtPls, in addition to giving us insight into what potential changes are needed. Based on these results, we will seek to build a pilot intervention among a subgroup of patients to enhance its adoption and sustained use.

**Figure:**
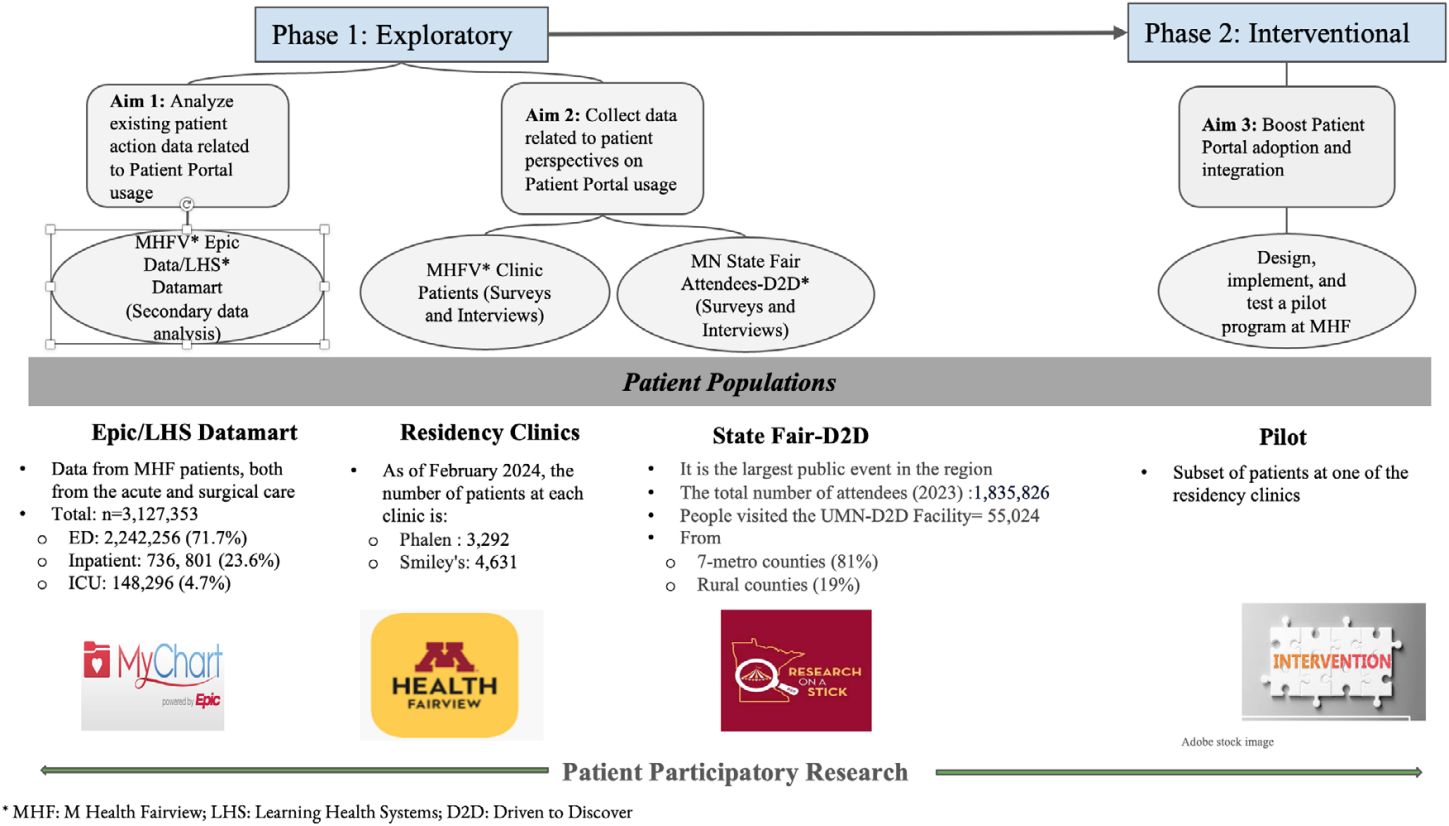
**FIGURE 1** Approach for understanding patient portal usage and testing strategies to enhance adoption.

## LEARNING BY DOING: TEACHING SURGERY RESIDENTS HOW TO CONDUCT A SYSTEMATIC REVIEW TO EVALUATE VENOUS THROMBOEMBOLISM PROPHYLAXIS PRACTICES IN COLORECTAL SURGERY

### Lauren Weaver MD^1^ *, Lindsay Welton MD^1^ *, Alexander Troester MD^1^ , Shelbi Olson MD^1^ , Julia Kohn^1^, MD, Sallee Brandt MPH^2^ , Bronwyn Southwell MD^2^ ^,3^, Mary Butler PhD, MBA^2^ , Paolo Goffredo, MD^4^

#### ^1^Department of Surgery, University of Minnesota, Minneapolis, MN, USA 55455; ^2^Evidence Synthesis Program, Center for Learning Health Systems Sciences, University of Minnesota, Minneapolis, MN, USA 55455; ^3^Department of Anesthesiology, University of Minnesota, Minneapolis, MN, USA; ^4^Division of Colon & Rectal Surgery, Department of Surgery, University of Minnesota, Minneapolis, MN, USA

*LW & LW equally contributed to this publication.

**Corresponding Author:** Lauren Weaver, weave500@umn.edu

**Introduction:** During medical training, residents are encouraged to utilize systematic reviews to facilitate their understanding and practice of evidence‐based medicine. However, there are limited opportunities for surgical trainees to cultivate the necessary skills to conduct these studies. Therefore, the University of Minnesota Evidence Synthesis Program (ESP) within the Center for Learning Health System and Sciences provides a unique opportunity to simultaneously learn and conduct a systematic review and meta‐analysis based on a research question of interest. In this feasibility case study, five general surgery residents, with no prior experience, learned how to conduct a systematic review and meta‐analysis to determine if the routine use of extended chemical venous thromboembolism (VTE) prophylaxis after abdominal colorectal surgery is supported by evidence within the literature.

**Methods:** To further explore this question, surgery residents (LW, AT, JK, LW, SO) and a colorectal surgeon (PG) met monthly with members of the ESP team (BS, SB, MB) to learn and conduct each step of the systematic review process. The motivating question was further refined by the Population, Intervention, Comparison, Outcome, and Time (PICOT) formula to develop keywords to aid a comprehensive literature review conducted by the web‐based PICO portal. Once the literature search was completed, key review questions pertaining to overall VTE rates among colorectal surgery patients and the effects of extended VTE prophylaxis for particular subgroups and types of anticoagulation were developed to assist with abstract screening. Abstracts were screened twice based on specific key questions and their corresponding inclusion and exclusion criteria. Screening discrepancies were resolved by a third party. The included abstracts were then screened as full‐text manuscripts following the same framework. Then manuscripts were assessed for risk of bias, and data were extracted.

**Results:** Our literature search pertaining to extended VTE prophylaxis in abdominal colorectal surgery yielded 831 abstracts that were screened for inclusion. A total of 91 abstracts underwent full‐text review. After screening, 4 randomized control trials were assessed for risk of bias using the Revised Cochrane risk‐of‐bias tool and 34 non‐randomized control trials were evaluated with the ROBINS‐I tool by two surgery residents. After identifying outcomes of interest, data were extracted from all manuscripts. Currently, the risk of bias assessment and data extraction are ongoing, but next steps will include evaluating manuscripts for strength of evidence.

**Conclusion:** This feasibility case study demonstrates the value of collaborating with evidence synthesis experts within a learning health system to equip surgical residents with the essential skills for conducting a systematic review and meta‐analysis. While mastering this process requires a significant amount of time and resources, trainees may leverage these skills to critically interpret the surgical literature and inform their surgical practice.

## MOTHER/INFANT OPIOID SUBSTANCE USE TREATMENT AND RECOVERY EFFORT (MOSTaRE) FAMILY EXPERIENCE SURVEY: A MULTI‐DISCIPLINARY, SYSTEMS‐LEVEL APPROACH TO QUALITY IMPROVEMENT

### Jessica Makori BA^1^, Jena Benson BA^1^, Alec Jonason BS^1^, Sénait Judge‐Yoakam BA^1^, Sonja Knudson BS^1^, Claudia LaRose BA, Tyler Radtke BA^1^, Susan Boehm BS, MS^2^, Jessica Cleghorn MPH^2^ , Gretchen Buchanan, PhD, LMFT, LADC^3^ ^,4^

#### ^1^ University of Minnesota Medical School, Minneapolis, Minnesota, USA; ^2^Minnesota Perinatal Quality Collaborative, Minnesota, USA; ^3^Redleaf Center for Family Healing, Hennepin Healthcare Research Institute, Minneapolis, Minnesota, USA; ^4^Department of Family Medicine and Community Health, University of Minnesota Medical School, Minneapolis, MN, USA

**Corresponding Author:** Gretchen Buchanan, gretchen.buchanan@hcmed.org

**Introduction:** The opioid crisis is a national and local public health concern. In Minnesota, over 1000 opioid‐related deaths occurred in 2022. Pregnant patients with substance use disorder (SUD) are among the most vulnerable. From 2018 to 2020, the SUD rate at delivery in Minnesota increased, peaking at 118.6 per 10 000 births. The Minnesota Perinatal Quality Collaborative (MNPQC) is a non‐profit organization that brings together various community stakeholders to improve perinatal health outcomes in MN. This session involved a panel discussion including the MNPQC executive director, staff, and members of their partnered research team to demonstrate a different approach to quality improvement and learning healthcare systems that goes beyond the walls of one healthcare system.

The Mother/Infant Opioid Substance Use Treatment and Recovery Effort (MOSTaRE) Initiative was developed utilizing the Alliance for Innovation on Maternal Health (AIM) safety bundle for the care of pregnant and postpartum people with substance use disorder (SUD). The initiative launched in September 2022 and emphasizes family‐centered care that maintains the maternal–infant dyad. It addressed treatment and prevention of substance exposure during pregnancy for mother/birthing person and infant.

To better inform the work being done by healthcare systems in the MOSTaRE learning collaborative, the MOSTaRE Family Experience Survey was developed to capture the voices of patients who experienced pregnancy and birth while using substances. Its results will help raise awareness among perinatal healthcare providers about mistreatment and bias and guide future quality improvement efforts across the state through MNPQC.

**Methods:** First, we assembled a workgroup including faculty from MNPQC Perinatal Equity Advisory Committee, the MOSTaRE Initiative Workgroup, medical students, and MNPQC staff. We then sought licensure and consultation from Birth Place Labs at the University of British Columbia to develop the survey using their validated Mothers on Respect index and their Mistreatment Index to assess respect in patient–provider interactions and person‐centered care. These surveys with supplemental questions were developed using Redcap, vetted by experts in the field and people with lived experience of the topic, and submitted to the IRBs of both the Hennepin Healthcare Research Institute and the University of Minnesota for QI/QA determination by the supporting researchers to ensure ethical use of the data. The final survey has predefined response options on a Likert scale with some open‐ended questions to allow participants to provide more detail. To capture the diversity of Minnesota, the survey and fliers were translated into Somali, Hmong, and Spanish.

**Results:** A pilot survey of 2–3 hospitals is currently underway and the survey will be released statewide in the coming weeks. Our goal is to reach at least 300 respondents statewide.

**Conclusion:** Our novel approach to quality improvement centers on a patient‐driven framework. MNPQC will use family experience survey results to collaborate with dozens of partner hospitals, analyze facility‐specific data, and create targeted action plans. The model prioritizes patient needs and feedback to improve outcomes and satisfaction. It also identifies inequities and biases faced by pregnant patients with SUD, guiding policy development to address these issues.

**Acknowledgements:** The authors would like to thank Christiana Johnson and the Birth Place Labs at the University of British Columbia.

## CO‐CREATING AN ADAPTATION AND IMPLEMENTATION PLAN FOR A NATIONAL EBP FOR PREGNANT AND PARENTING PEOPLE WITH OPIOID USE DISORDER

### Gretchen Buchanan, PhD, LMFT, LADC^1^ ^,2^ Biftu Abdullahi, MPH,^1^ Tori Simenec, MA,^4^ Helen Kim, MD^1^ ^,3^

#### ^1^ Redleaf Center for Family Healing, Hennepin Healthcare System; ^2^University of Minnesota Medical School, ^3^University of Minnesota Department of Family Medicine and Community Health; ^4^University of Minnesota Department of Psychiatry; ^4^ Institute for Child Development

**Corresponding Author:** Gretchen Buchanan, gretchen.buchanan@hcmed.org

**Introduction**: Opioid use disorder among pregnant and parenting individuals is a growing public health concern. Treatment for this population is fragmented and difficult to access, meaning many never receive treatment or drop out. Meanwhile, healthcare systems lack enough trained staff and appropriate processes and protocols. The Alliance on Innovation in Maternal Health's (AIM) Care for Pregnant and Postpartum People with Substance Use Disorder (CPPPSUD) Patient Safety Bundle is supported by HRSA and is being disseminated nationally, but research supporting best implementation strategies is needed. The current study, a formative evaluation, is the first step in adapting CPPPSUD bundle to a local context through an organized implementation process (see Figure 1 below).

**Methods:** Using rapid qualitative analysis, we conducted key informant interviews with three groups of people impacted by the change: Hennepin Healthcare staff (*n* = 19), staff at external organizations serving the population (*n* = 16), and patients (*n* = 5). We evaluated current resources and gaps in care, treatment priorities, and feasibility, acceptability, and appropriateness of proposed changes to current services. Staff and patients also completed surveys that identified the presence and importance of the 17 components of the CPPPSUD bundle and a baseline validated quality care measure. The second step refined details of the model and plan with the same participants.

**Results:** We identified barriers and facilitators using the PRISM framework and potential ERIC+ implementation strategies and potential members of the clinical and implementation team and community advisory board. There are many services available locally for people struggling with substance use disorders, but care is fragmented internally and externally. Participants emphasized the need for system‐level changes; it was not enough to simply offer services, but to change healthcare and social culture of stigma and shame for this population. Physical structures and clinical set‐ups are not ideal and need to change to accommodate the needs of both healthcare workers and patients. Intricately interwoven was the importance of culturally sensitive care and hiring a diverse workforce to support a sense of belonging for diverse patients. Patients were drawn to programming that specifically addressed their roles as parents and provided support for bonding and attachment. Patients reported prioritizing their sobriety and children and recognized a desire to be in recovery as key to treatment engagement. Employees and external organizations in the interviews also highlighted complexity and cost burden, challenges with reimbursement, and strategies for sustaining the program. The foundational services will be established first, along with systemic changes including workflows, physical structures, and staff training to support a positive culture of recovery for this population. We have partnered with EMS to develop the EMS‐based Recovery and Community Engagement (EMBRACE) Program to provide enhanced support outside the hospital setting. This initiative offers immediate access to medication and referral to the Redleaf Center for Family Healing and the Addiction Medicine Clinic.

**Conclusion:** Through an iterative co‐design process, we operationalized and adapted the AIM CPPPSUD bundle and developed a staged implementation plan. A dynamic implementation will occur over the next 5 years followed by an RCT through a statewide network of perinatal healthcare providers.

**Figure:**
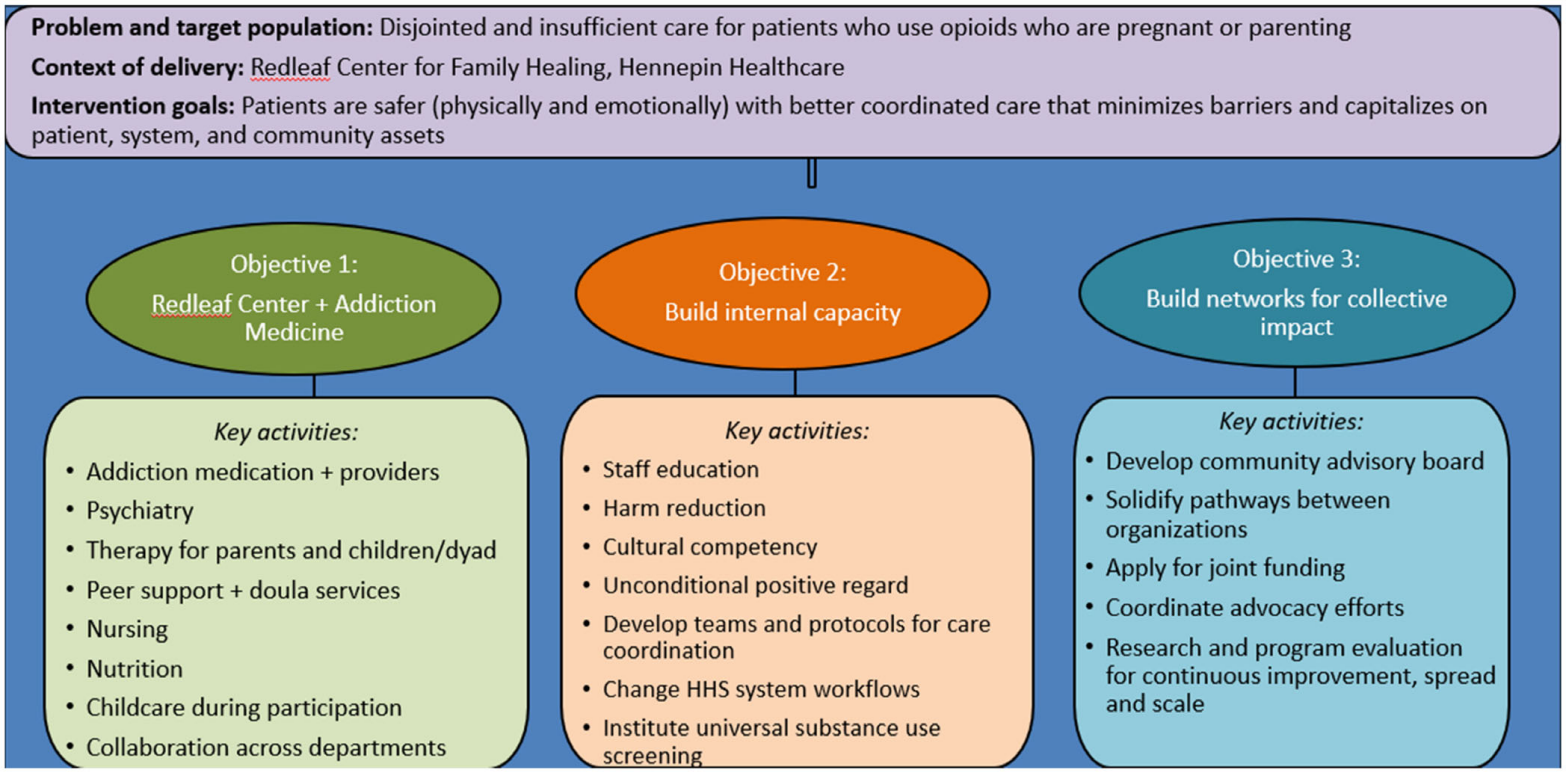
**FIGURE 1**.

**Acknowledgements:** We would like to thank all the patients, staff, and external employees who took the time to talk with us during our evaluation.

## USING NATIONAL ORGAN DONATION DATA TO INFORM LOCAL ORGAN PROCUREMENT ORGANIZATION COMMUNITY ENGAGEMENT

### J.M. Miller^1^, N. Salaam^2^, W.T. McKinney^1^ ^,3^, J. McWilliams^1^ , B. Martinez^2^, P. St. John^2^, J. Azure^5^, T. Wyatt^4^, S. Mau Larson^2^, C.R. Schaffhausen^1,3 *^

#### ^1^ Hennepin Healthcare Research Institute (HHRI), Minneapolis, MN; ^2^ LifeSource, Minneapolis, MN; ^3^ University of Minnesota (UMN), Minneapolis, MN; ^4^ Hennepin Healthcare, Minneapolis, MN; ^5^ Community partner, Belcourt, ND

**Corresponding author:** Cory Schaffhausen, schaf390@umn.edu

**Introduction:** Organ Procurement Organizations (OPOs) are non‐profit organizations that manage organ donation in a designated donation service area (DSA). To increase the availability of organs from deceased donors, to meet regulatory standards, and provide a transparent and equitable donation system, OPOs need to be able to track interventions and changes in practice across their geography. Currently, disparities in organ transplant persist throughout the transplant process, in part due to medical mistrust and barriers in access. This may mean that communities with a higher need for transplants also have lower rates of organ donation. Individual OPOs track organ donation data and provide data to national registries such as the Organ Procurement and Transplantation Network (OPTN); however, these data have not been previously used at a national scale to promote embedded research and improve equity in organ donation. This study describes the development of a tool that allows OPOs to track their donor recovery performance across time and geography in their DSA. LifeSource, the OPO serving Minnesota and surrounding states, is a partner and early adopter in the planning, development, and testing for organ donation data tools.

**Methods:** Unadjusted and adjusted donation rates were calculated for each county in the US. The numerator for these rates was the number of donors with at least one organ transplanted as reported to the OPTN. The denominator was the number of Cause, Age, and Location Consistent with donation (CALC) deaths as enumerated in the Centers for Disease Control and Prevention (CDC) Multiple Cause of Death dataset. Adjusted donation rate ratios were adjusted for age, sex, and race/ethnicity. Results are displayed on an interactive map using R Shiny.

**Results:** Among counties with 10 or more provisional CALC deaths in 2023, the unadjusted donation rate ranged from 0 to 50 donors per 100 CALC deaths. The interactive map shows donation rates by county with green indicating higher donation rates and red indicating lower donation rates (Figure 1). OPOs can additionally explore different years from 2020 to the first 2 months of 2024 and can explore adjusted donation rate ratios by age, sex, race, and ethnicity.

**Conclusion:** This tool can help OPOs identify areas for intervention to improve their donation rates and can be used to evaluate the effectiveness of such interventions across time and geography. Local data can be a resource to help engage communities and to improve transparency about the need for organ donation within the community. Future work will seek broad partner and user input to refine the data presentations.

**Figure:**
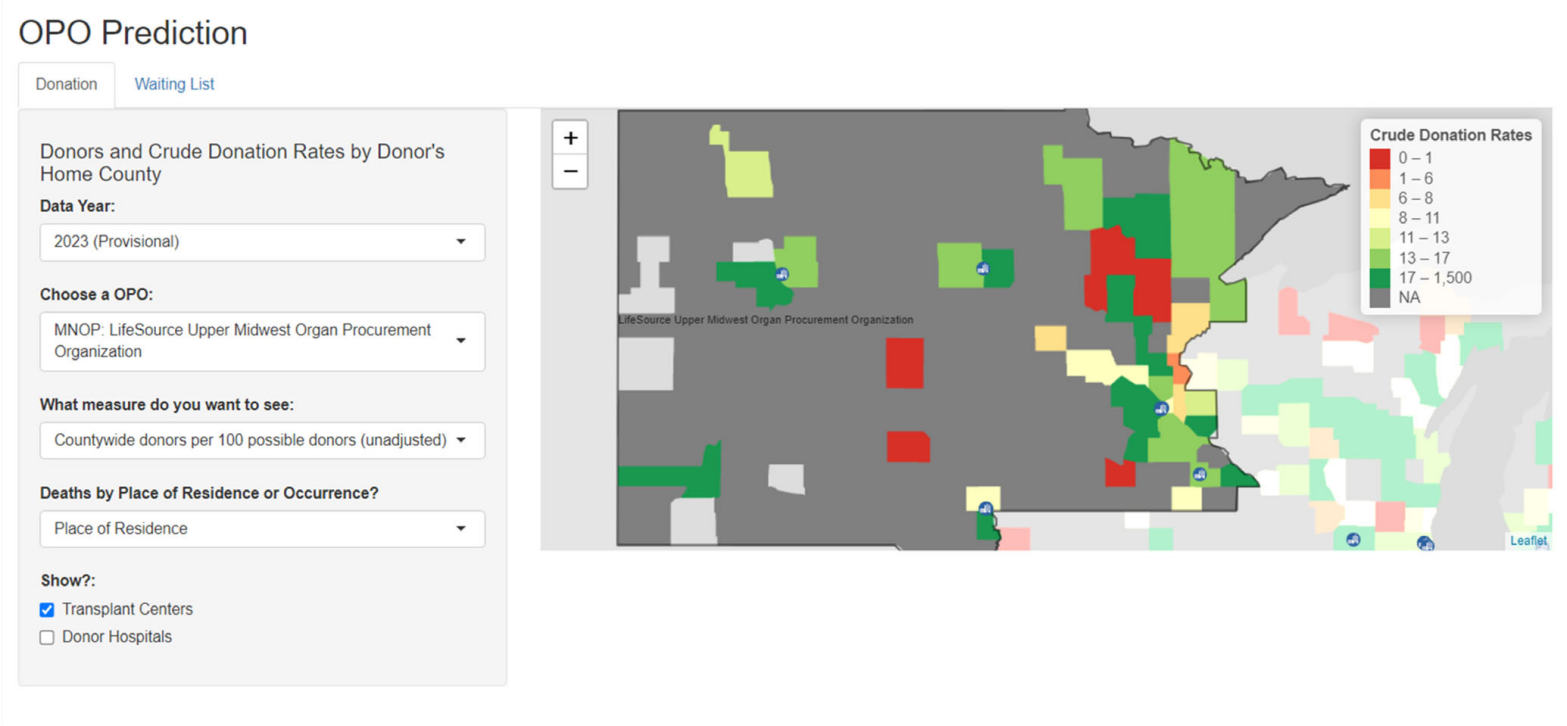
**FIGURE 1** Unadjusted donation rates by county in 2023 in the Upper Midwest.

## TARGETED VERSUS HIGH‐INTENSITY MONITORING FOLLOWING INTRAVENOUS THROMBOLYSIS IN ACUTE ISCHEMIC STROKE^A^

### Carl‐Lewis Valcinord^1^ MD, Rebecca Roberts^1^ MD, Sohum Bindra^1^, Marcus Milani^1^, Abbey Staugaitis^1^ MSN, RN, Megan Tessmer^1^ BSN, RN, Jodi Mueller‐Hussein^1^ BSN, RN, Christopher Streib^1^, MD, MS

#### ^**1**^ University of Minnesota, Twin Cities, Minneapolis, MN USA

**Corresponding author:** Carl‐Lewis Valcinord, cvalcino@umn.edu

**Introduction:** Intravenous thrombolysis (IVT) is the only FDA‐approved medical treatment for acute ischemic stroke (AIS). However, IVT is associated with severe complications such as symptomatic intracranial hemorrhage (sICH). Therefore, current guidelines recommend 24‐hour high‐intensity monitoring (HIM) in an intensive care unit (ICU) for all AIS patients post‐IVT. This practice is resource‐intensive and of unclear utility in patients with mild to moderate AIS. We studied a targeted‐intensity monitoring (TIM) protocol for low‐risk AIS patients following IVT.

**Methods:** Post‐IVT patients were considered low risk if their blood pressure was <180/105, their level of consciousness was preserved, no high‐risk vessel stenosis or occlusion was present on imaging and NIHSS ≤10. The NIHSS is a stroke severity score ranging from 0 to 42. All patients meeting these criteria from October 2020 to April 2024 were included in our study. The TIM pathway was implemented in our institution in March 2022, and eligible post‐IVT AIS patients were monitored in an intermediate care unit (IMC) with neurological exams and vital sign assessments every 15 minutes for the first hour, every hour for the next 3 hours, every 2 h for the next 8 h, and every 4 h for the final 12 h, totaling 14 neurological assessments and vital sign measurements over 24 h. Post‐IV AIS patients who presented prior to the implementation utilized the conventional HIM pathway and were monitored in the ICU with neurological assessments and vital sign (VS) measurements every 15 minutes for the first 2 hours, every 30 minutes for the next 6 h, and then every hour thereafter, totaling 36 neurological and vital sign assessments over 24 h. We examined the frequency TIM patients were transferred to the ICU, ICU length of stay, hospital length of stay, symptomatic intracranial hemorrhage (sICH), NIHSS at 24 h, and early neurologic deterioration (END [NIHSS increase ≥4]). Statistical testing was performed using chi‐squared test and two‐sample t‐test.

**Results:** 95 patients were included: 47 HIM (median age 71 [IQR 56–75.5], median NIHSS 4) and 48 TIM (median age 65, [IQR 60–81.25], median NIHSS 4). The mean ICU stay for HIM was 32.8 h; no TIM patients were transferred from IMC to ICU. There was no difference in sICH rate: HIM 2.1% vs. TIM 0% (*p* = 0.31); median hospital length of stay: HIM 49.8 hours (IQR: 43.8–83.3) vs. TIM 49.6 hours (IQR: 32.6–99.7) (*p* = 0.72); median NIHSS at 24 hours: HIM 2 (IQR: 0–3.5) vs. TIM 1 (IQR: 0–2) (*p* = 0.42); or early neurological decline: HIM 0% vs. TIM: 2.1% (*p* = 0.32).

**Conclusion:** A targeted monitoring approach for post‐IVT AIS patients was safe and feasible in selected patients. Post‐IVT TIM pathways may conserve healthcare resources and increase ICU bed availability. However, further studies defining the optimal post‐IVT TIM criteria are indicated.

- Awarded best poster submission of the 2024 AiLHHS Conference

## CONFRONTING UNCERTAINTY AND ADDRESSING URGENCY FOR ACTION THROUGH THE ESTABLISHMENT OF A VA LONG COVID PRACTICE BASED RESEARCH NETWORK

### Alicia B. Woodward‐Abel, MPH^1^ ; Carla Amundson, MA^1^ ; Emily Hudson, MS^1^ ; Troy Layouni, MPH^2^ ; Sena Soleimannejad, MPH^3^ ; Megan Miller, PhD^2^ ^,4^; Collin Calvert, PhD, MPH^1^ ^,5^; Tammy L. Eaton, PhD, MSc, FNP‐BC^6^ ^,7,8^; Norbert Bräu, MD, MBA^3^ ^,9^; Kristina Crothers, MD^2^ ^,10,11^; R. Adams Dudley, MD, MBA^1^ ^, 5^; Aaron P Turner, PhD^2^ ^,4^; Allison M. Gustavson, PT, DPT, PhD^1^ ^,12,5,13^

#### ^1^Veterans Affairs Center for Care Delivery and Outcomes Research, Minneapolis VA Health Care System, Minneapolis, MN, USA; ^2^VA Puget Sound Health Care System, Seattle, WA USA; ^3^James J. Peters VA Medical Center, Bronx, NY; ^4^Department of Rehabilitation Medicine, University of Washington, Seattle, WA 98115 USA; ^5^Department of Medicine, University of Minnesota, Minneapolis, MN 55455 USA; ^6^Department of Internal Medicine, Division of Hospital Medicine, Michigan Medicine, Ann Arbor, MI, USA; ^7^VA Center for Clinical Management Research, VA Ann Arbor Healthcare System, Ann Arbor, MI, USA; ^8^Institute for Healthcare Policy and Innovation, University of Michigan, Ann Arbor, MI, USA; ^9^Icahn School of Medicine at Mount Sinai, New York, New York; ^10^Seattle‐Denver Center of Innovation (COIN) for Veteran‐Centered and Value‐Driven Care; ^11^Department of Medicine, University of Washington, Seattle, WA 98115 USA; ^12^Veterans Affairs Rehabilitation Research and Development Center for Rehabilitation & Engineering Center for Optimizing Veteran Engagement & Reintegration, Minneapolis Veterans Affairs Health Care System, Minneapolis, MN 55417 USA; ^13^Department of Family Medicine and Community Health, Rehabilitation Sciences Division, University of Minnesota, Minneapolis, MN 55455 USA

**Corresponding Author:** Alicia B. Woodward‐Abel, MPH, Minneapolis Veterans Affairs Health Care System, 1 Veterans Drive, Mail Code #152, Minneapolis, MN 55417, Alicia.Woodward-Abel@va.gov

**Introduction:** Learning Health Systems (LHS) show promise in making sense of uncertainty and emerging evidence in complex conditions such as Long COVID. Long COVID is defined by the ongoing, new, or returning symptoms following COVID‐19 infection that negatively impacts return to meaningful participation in social, recreational, and vocational activities. The interdisciplinary, clinical uncertainty surrounding Long COVID is amplified by unclear etiology and unknown prognosis. Long COVID is an example of a condition that challenges how we deliver care due to the uncertainty surrounding best clinical practices, processes, and policies for Long COVID care that have resulted in practice variation despite the emerging, though limited, evidence base. Failure to address gaps in clinical evidence and implementation of care pathways threatens to perpetuate fragmented, burdensome, and unnecessary care.

**Methods:** The context surrounding Long COVID created an urgency to make sense of uncertainty and rapidly address urgent, clinically relevant questions. Thus, the Veterans Health Administration (VHA) funded a Long COVID Practice Based Research Network (LC‐PBRN) to build an infrastructure that would support communication and coordination within the larger Long COVID ecosystem. In doing so, the vision of the LC‐PBRN is to centralize Long COVID clinical, research, and operational activities across the VA to ensure shareholder needs are identified, resources are allocated appropriately, and redundancy in efforts is avoided. The research infrastructure of the LC‐PBRN is designed with a Learning Health System (LHS) lens to facilitate feedback loops necessary to make progress towards our vision.

**Results:** Throughout the process of establishing the LC‐PBRN infrastructure, we identified several important lessons learned: (1) include Veterans' voices to ensure network efforts address and align with patient needs, (2) develop an interdisciplinary leadership team to foster the diverse viewpoints needed to tackle multifaceted problems, (3) set clear expectations and goals with partners, (4) build engaging relationships to bridge any gaps between partners within and outside the VHA, and (5) establish and maintain clear internal deliverables and timeline management to coordinate a complex network.

**Conclusions:** This work informs the evolving role of PBRNs as an integral part of building and sustaining LHS infrastructure.

## PERIOPERATIVE COLORECTAL SURGERY NUTRITION SYSTEMATIC REVIEW: A USE CASE OF THE EVIDENCE SYNTHESIS PROGRAM

### Alexander Troester, MD^1^ , Shelbi Olson, MD^1^ , Lindsay Welton, MD^1^ , Lauren Weaver, MD^1^ , Bronwyn Southwell, MD^2^ , Sallee Brandt, MPH^3^ , Mary Butler, PhD, MBA^3^ , Paolo Goffredo, MD^1^

#### ^1^ Division of Colon & Rectal Surgery, Department of Surgery, University of Minnesota, Minneapolis, MN, USA; ^2^ Department of Anesthesia, University of Minnesota, Minneapolis, MN, USA; ^3^ School of Public Health, University of Minnesota, Minneapolis, MN, USA

**Corresponding Author**: Paolo Goffredo, MD, 420 Delaware St. SE, MMC 450, Minneapolis, MN 55455, Email: goffr002@umn.edu, Twitter handles: @GoffredoPaolo, @UMNSurgery, Tel: 612–625‐7992; Fax: 612–625‐4406.

**Introduction:** Enhanced recovery after surgery (ERAS) contains multiple components intended to optimize patient outcomes. Perioperative nutritional interventions in colorectal ERAS pathways are heterogenous and poorly defined. We partnered with the Evidence Synthesis Program within the Center for Learning Health System Sciences to gain hands‐on training as clinicians in conducting a methodologically rigorous systematic review with our findings regarding perioperative colorectal nutrition strategies being directly translational to patient‐centered care.

**Methods:** Four general surgery residents interested in colorectal surgery underwent training in Risk of Bias and Strength of Evidence assessment from members of the Evidence Synthesis Program during monthly meetings. Concurrently, a comprehensive literature search was conducted and the web‐based screening tool PICO Portal™ was utilized to screen citations at abstract and full‐text level. Data extraction for all studies, independent of risk of bias assessment was performed.

**Results:** A total of 5041 abstracts were screened, with 118 undergoing full‐text review, and 27 studies ultimately meeting inclusion criteria. Of these, 19 were randomized controlled trials and 8 were non‐randomized studies of interventions. Risk of bias assessment was low in 5 studies, some in 5 studies, and high in 9 studies. All outcomes of interest were rated as either insufficient or low strength of evidence, frequently citing high study limitations and imprecise estimates as the underlying reason.

**Conclusions:** We identified perioperative colorectal nutrition recommendations as a topic warranting in‐depth investigation to better inform clinical practices. As clinicians, critical appraisal of the literature is viewed through the lens of practical application at the bedside. Using this perspective, and partnering with experts in evidence synthesis, learning health systems can efficiently and effectively utilize the clinician's viewpoint in order to continually improve patient care by conducting methodologically rigorous systematic reviews on selected topics. These findings have the potential to inform internal colorectal surgery perioperative guidelines through specific care maps.

